# The Emerging Role of tRNA-Derived Small RNAs (tsRNAs) in Radiation-Induced Cardiovascular Pathology

**DOI:** 10.2174/0118715303460374260127095032

**Published:** 2026-03-30

**Authors:** Ying Chen, Yunjia Zhu, Lingmei Qian, Qunchao Hu

**Affiliations:** 1 Hongqiao International Institute of Medicine, Tongren Hospital, Shanghai Jiao Tong University School of Medicine, Shanghai, 200336, China;; 2 Department of Radiation Oncology, Tongren Hospital, Shanghai Jiao Tong University School of Medicine, Shanghai, 200336, China

**Keywords:** tRNA-derived small RNAs, oxidative stress, fibrosis, inflammation, endothelial dysfunction, radiotherapy

## Abstract

**Introduction:**

Ionizing radiation exposure during thoracic radiotherapy is a risk factor for long-term cardiovascular morbidity, including radiation-induced heart disease (RIHD). Emerging evidence implicates transfer RNA-derived small RNAs (tsRNAs) as stress-responsive regulatory molecules, yet their roles in RIHD remain unexplored.

**Methods:**

A comprehensive literature review was conducted to explore tsRNA biology, radiation-induced cardiovascular injury, and non-coding RNA function. We systematically searched databases including PubMed, Web of Science, and Scopus using keywords such as “tsRNA,” “tRNA-derived fragments,” “radiation-induced heart disease,” “oxidative stress,” and “cardiovascular injury.” Literature screening prioritized original research articles and high-impact reviews providing mechanistic insights into tsRNA expression, biogenesis, or function in the context of radiation response or cardiovascular pathologies. Non-peer-reviewed articles, conference abstracts, non-English publications, or studies not directly relevant to the core themes were excluded. The selected studies were analyzed to identify and synthesize the mechanistic links between tsRNA dysregulation and radiation-induced cardiovascular damage.

**Results:**

Ionizing radiation robustly induces tsRNA biogenesis *via* endonucleases (*e.g*., angiogenin, Dicer) in cardiovascular cells. Post-irradiation, specific tsRNAs are dysregulated and contribute to key RIHD mechanisms, including sustained oxidative stress, mitochondrial dysfunction, endothelial senescence, DNA damage response, and fibrotic remodeling. These tsRNAs modulate critical signaling pathways, including PI3K/AKT, JNK, and NF-κB, thereby regulating targets involved in redox balance, apoptosis, and extracellular matrix deposition. Notably, circulating tsRNAs emerge as promising early, non-invasive biomarkers of radiation exposure in both preclinical and clinical settings, highlighting their translational potential.

**Discussion:**

Our study reveals that tsRNAs serve as molecular transducers linking acute radiation stress to chronic cardiovascular dysfunction. Further, they hold promise as early warning biomarkers and therapeutic targets in cardio-oncology.

**Conclusion:**

RIHD is pathologized by tsRNAs. They identify potential non-invasive biomarkers to detect RIHD early and possibly target it for treatment in cancer survivors.

## 1 INTRODUCTION

Ionizing radiation exposure, particularly in thoracic radiotherapy for cancers such as breast cancer and Hodgkin lymphoma, is increasingly recognized as a significant contributor to long-term cardiovascular morbidity [1]. Clinical and epidemiological studies have consistently demonstrated an elevated risk of radiation-induced heart disease (RIHD) [2], manifesting as coronary artery disease, myocardial fibrosis, valvular dysfunction, and pericarditis years to decades post-treatment [3]. The mechanisms underlying radiation-induced cardiovascular injury are complex and multifactorial, involving DNA damage, sustained oxidative stress, chronic low-grade inflammation, microvascular rarefaction, and activation of fibrotic pathways [4]. Critically, these pathological processes constitute a “hidden threat” that can progress long after radiation exposure ends. At present, we still lack early biomarkers for the prevention and targeted therapeutic interventions for RIHD [5].

In recent years, an increasing number of functional non-coding RNAs have emerged as critical regulators of cardiovascular homeostasis and disease [6]. Among these, tsRNAs, including tRNA-derived fragment-5 (tRF-5), tRF-3, tRF-1, tRF-2, and i-tRFs (14-32 nt in length), have been recognized as a novel class of functional regulatory RNAs [7]. Traditionally viewed as inert cleavage products of tRNAs, tsRNAs are generated in a regulated manner under stress conditions (*e.g*., UV irradiation, hypoxia, and oxidative stress) by ribonucleases such as angiogenin (ANG), Dicer, RNase Z, and RNase P [8]. Studies have revealed that tsRNAs are not degradation byproducts but evolutionarily conserved molecules capable of modulating gene expression, translation, and signal transduction through interactions with RNA-binding proteins, argonaute complexes, or target mRNAs [9, 10]. Despite growing evidence of tsRNA involvement in cancer and neurodegenerative diseases [10, 11], their role in radiation biology and cardiovascular pathology remains largely unexplored. Given that radiation is a potent inducer of cellular stress and, consequently, tsRNA biogenesis, it is plausible that tsRNAs serve as key molecular mediators linking radiation exposure to long-term cardiovascular damage [12].

This systematic narrative review integrated current knowledge on tsRNA biology to elucidate their potential roles in the pathogenesis of RIHD, with a particular focus on their utility as early biomarkers and novel therapeutic targets. To ensure comprehensive coverage, we conducted a structured literature search across PubMed, Web of Science, and Scopus up to August 2025. Search terms included combinations of keywords related to tsRNAs (*e.g*., “tsRNA,” “tRNA-derived fragment,” “tRF,” “tiRNA”), ionizing radiation (“radiotherapy,” “radiation exposure”), and cardiovascular pathology (“oxidative stress,” “fibrosis,” “endothelial dysfunction,” “heart disease”). Original research articles and high-impact reviews providing mechanistic insights into tsRNA biogenesis, function, or biomarker potential in the context of radiation or cardiovascular stress were prioritized. Selected studies were thematically synthesized to construct a coherent framework linking radiation-induced tsRNA dysregulation to the molecular pathways of chronic cardiac injury. This review aimed to provide a novel perspective on the molecular mechanisms of RIHD and to lay a theoretical foundation for the development of early detection and targeted interventions.

## 2 BIOGENESIS AND CLASSIFICATION OF TSRNAS

**Fig. (1):**
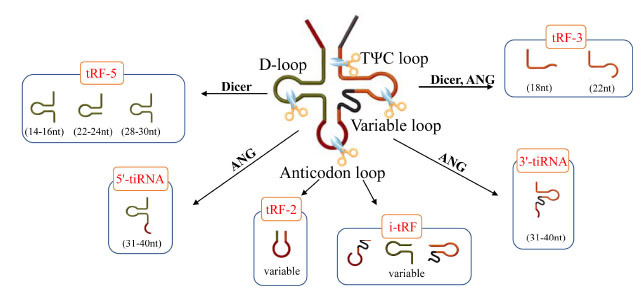
Biogenesis and classification of tRNA-derived small RNAs (tsRNAs) under stress conditions. This schematic illustrates the canonical cloverleaf secondary structure of a mature transfer RNA (tRNA) and its cleavage by specific ribonucleases under cellular stress, leading to the generation of distinct tsRNA subtypes.

Once regarded as degradation byproducts, tsRNAs are now recognized as functionally diverse regulatory molecules whose biogenesis is tightly controlled and context-dependent. The canonical cloverleaf structure of mature tRNA contains several conserved regions, including four loops (D-loop, TΨC loop, variable loop, anticodon loop) and four arms [13]. Under cellular stress such as oxidative stress, hypoxia, ionizing radiation, or inflammation, specific endonucleases cleave tRNAs at precise sites, generating distinct tsRNA subtypes (Fig. **1**). The primary enzymes involved in tsRNA generation include ANG, Dicer, RNase Z, and RNase P, each with distinct cleavage preferences. Based on their origin and biogenesis pathways, tsRNAs are broadly classified into the following subtypes:

- tRF-5 and tRF-3: Derived from the 5′ or 3′ ends of mature tRNAs, respectively, these fragments are primarily generated in a Dicer-dependent manner. They often retain partial sequence complementarity to the parental tRNA and can function similarly to miRNAs by loading onto AGO proteins to regulate mRNA stability and translation [14].
- tRF-1: This subtype originates from the 3′ trailer sequence of precursor tRNAs (pre-tRNAs), which is cleaved by RNase Z during tRNA maturation, and tRF-1 fragments are therefore unique to the processing of pre-tRNAs and are often upregulated in disease states, including cancer [15].
- tiRNA (tRNA-derived stress-induced RNA) or tRNA half: These are generated by angiogenin-mediated cleavage in the anticodon loop of mature tRNAs under oxidative stress and other cytotoxic conditions, including ionizing radiation [16]. Typically 30–40 nt in length, tiRNAs do not typically associate with AGO but may interact with RNA-binding proteins to modulate stress granule formation, and apoptosis [17, 18].

## 3 RADIATION-INDUCED CARDIOVASCULAR INJURY: KEY MECHANISMS

Radiation therapy is a crucial treatment for treating thoracic malignancies such as breast cancer, Hodgkin’s lymphoma, and lung cancer [19]. However, incidental exposure of the heart and great vessels to ionizing radiation can cause delayed and progressive cardiovascular damage, collectively known as RIHD [20, 21]. This condition encompasses pericarditis, microvascular dysfunction, accelerated atherosclerosis, myocardial fibrosis, valvular calcification, and conduction disorders. These complications may manifest years after treatment and represent a major long-term concern for cancer survivors [22, 23]. The pathogenesis of RIHD is multifactorial, involving sustained oxidative stress, chronic inflammation, endothelial dysfunction, DNA damage, and aberrant tissue remodeling. Below, we outlined the key cellular and molecular mechanisms underlying radiation-induced cardiovascular injury.

### 3.1 Endothelial Dysfunction and Microvascular Damage

The vascular endothelium is a monolayer of endothelial cells lining blood vessels that acts as a dynamic barrier and sensor for hemodynamic, hormonal, and biochemical signals, and is highly sensitive to ionizing radiation [24]. Acute radiation exposure can induce endothelial cell apoptosis, senescence, and barrier disruption, promoting vascular leakage and microthrombosis [25]. NO, produced by endothelial nitric oxide synthase (eNOS), is a key mediator that regulates vascular tone to meet cellular oxygen requirements [26]. Specifically, NO induces smooth muscle relaxation *via* cGMP-dependent activation of protein kinase G (PKG) while also inhibiting platelet aggregation through cAMP elevation, thereby preventing thrombosis [27, 28]. Endothelial dysfunction, characterized by reduced NO bioavailability, is an early hallmark of cardiovascular disease [29]. This dysfunction disrupts the balance between vasodilators (*e.g*., NO, prostacyclin) and vasoconstrictors (*e.g*., endothelin-1, angiotensin II), promoting vasoconstriction, vascular leakage, and leukocyte adhesion [30]. Pro-inflammatory mediators such as IL-1α, IL-1β, IL-6, TNF-α, and VEGF further drive endothelial activation, leading to a pro-adhesive and pro-thrombotic state [31]. Infiltrating immune cells, particularly M1-polarized macrophages, amplify the inflammatory response and release mediators that drive fibrosis and tissue remodeling [32].

In addition to regulating vascular tone and inflammation, ECs are central to angiogenesis. Following injury or hypoxia, they transition from a quiescent to an activated, proliferative state to support repair. However, dysregulated angiogenesis contributes to microvascular rarefaction, tissue hypoperfusion, and end-organ damage [33]. Radiation could significantly induce DNA damage and oxidative stress in endothelial cells, triggering senescence and cell death. In vascular injury, tiRNA-Glu-CTC promotes damage by inducing mitochondrial dysfunction [34].

### 3.2 Oxidative Stress and Mitochondrial Dysfunction

Ionizing radiation-induced oxidative stress and consequent mitochondrial dysfunction are not merely downstream consequences but serve as primary and sustained drivers that initiate and amplify multiple pathological cascades in RIHD. The radiolysis of water directly generates a burst of reactive oxygen species (ROS), such as superoxide anion (O_2_^−^•) and hydroxyl radicals (•OH), which can acutely overwhelm endogenous antioxidant defenses (*e.g*., SOD, GPX4) [35]. Crucially, ROS function as persistent signaling molecules that propagate damage beyond initial oxidation. They activate key stress-sensitive pathways, including NF-κB and MAPK (*e.g*., JNK) cascades, thereby bridging oxidative stress to chronic inflammation and cell death signaling [36]. Mitochondria are both significant sources of and critical targets for radiation-induced ROS, creating a vicious cycle of dysfunction. Radiation directly impairs the electron transport chain, leading to loss of mitochondrial membrane potential, reduced ATP production, and increased leakage of pro-apoptotic factors like cytochrome c [37]. A particularly vulnerable target is mitochondrial DNA (mtDNA), which, due to its proximity to the ROS source and lack of histone protection, suffers disproportionate damage. The release of damaged mtDNA into the cytosol can activate the cGAS-STING innate immune pathway, fueling a sterile inflammatory response that exacerbates tissue injury [38].

The dysregulation of mitochondrial quality control (MQC) mechanisms is central to sustaining this dysfunctional state. Mitochondrial dynamics, the balance between fission and fusion, are crucial for maintaining a healthy mitochondrial network. Radiation disrupts this balance, often promoting excessive fission, which facilitates the segregation and subsequent clearance of damaged mitochondria *via* mitophagy [39]. Importantly, this process is intricately linked to cellular energy sensing. The mTOR system is a master regulator that integrates nutrient and stress signals to control cell growth and autophagy. Under radiation stress, inhibited mTORC1 activity relieves its suppression of the ULK1 complex, a key initiator of autophagosome formation, thereby promoting mitophagy [40]. The PI3K pathway, upstream of mTOR and its effector AKT, plays a particularly important role in regulating this autophagic response to oxidative and genotoxic stress [41]. Concurrently, radiation can suppress mitochondrial biogenesis, governed by master regulators like PGC-1α, which impairs the replacement of damaged mitochondrial networks and compromising cardiac energetics [42]. This multifaceted failure of MQC perpetuates oxidative stress and bioenergetic deficit in cardiomyocytes.

These oxidative and mitochondrial insults directly drive multiple RIHD pathologies. In endothelial cells, sustained ROS production contributes to dysfunction by reducing NO bioavailability and promoting a pro-adhesive, pro-thrombotic state [24, 37]. In the vasculature, oxidative stress triggers lipid peroxidation and modifies lipoproteins, facilitating lipid retention and VSMC phenotypic switching, which accelerates atherosclerotic plaque development [43]. At the cardiomyocyte level, radiation-induced suppression of protective factors like SIRT3 disrupts redox balance (as evidenced by elevated ROS and MDA, and decreased SOD/GSH), leading to energetic failure and contractile dysfunction [44]. Furthermore, oxidative stress and mitochondrial dysfunction potently induce cellular senescence and senescence-associated secretory phenotype (SASP), creating a pro-fibrotic microenvironment [45]. Triggered by chronic oxidative stress and inflammatory cues *via* PI3K and other pathways, the sustained activation of AKT signaling plays a pivotal role in cardiac fibrosis. It activates cardiac fibroblasts, promoting their proliferation and transition to collagen-secreting myofibroblasts, thereby driving late cardiac fibrotic remodeling [46].

Thus, oxidative stress and mitochondrial dysfunction act as a critical pathogenic nexus in RIHD. They are early, persistent events that not only directly inflict cellular damage but also, through broad signaling rewiring (*e.g*., *via* the mTOR/PI3K/AKT axis), activate and interconnect the inflammatory, fibrotic, and senescent pathways. These interconnected pathways characterize chronic radiation-induced cardiovascular injury.

### 3.3 Endoplasmic Reticulum (ER) Stress, mTOR Inhibition, and Autophagy

Beyond direct genomic and mitochondrial targeting, ionizing radiation disrupts proteostasis by inducing protein misfolding and aggregation within the ER, triggering the unfolded protein response (UPR) [47]. The UPR, mediated by sensors like PERK, IRE1α, and ATF6, initially aims to restore ER homeostasis. However, sustained radiation-induced ER stress drives pathological outcomes central to RIHD. A pivotal mechanism involves the PERK-eIF2α axis. Upon activation, PERK phosphorylates eukaryotic initiation factor 2α (eIF2α), leading to a global but transient attenuation of protein synthesis to reduce the ER burden [48]. Importantly, this phosphorylation also specifically inhibits the mechanistic target of rapamycin complex 1 (mTORC1), a master regulator of cell growth, metabolism, and survival [49]. The suppression of mTORC1 serves as a critical switch to activate autophagy, a catabolic process for degrading damaged organelles and proteins [40]. Under moderate radiation stress, this ER stress-mTOR-autophagy axis may function as a cytoprotective response, clearing dysfunctional components. However, under the high-dose or chronic exposure typical of thoracic radiotherapy, the response becomes maladaptive. Persistent ER stress and excessive autophagy contribute to endothelial cell apoptosis and cardiomyocyte loss [50]. Furthermore, chronic mTOR inhibition and autophagic flux alterations in cardiac fibroblasts can promote their activation and increase the secretion of extracellular matrix proteins, thereby exacerbating myocardial fibrotic remodeling [51]. Notably, the ER stress and UPR pathways exhibit extensive crosstalk with oxidative stress and inflammatory signaling (*e.g*., *via* NF-κB), creating a feed-forward loop that amplifies tissue injury and promotes the SASP [38]. Thus, the ER stress-mTOR-autophagy axis represents a fundamental yet underappreciated mechanism that transduces radiation-induced proteotoxic stress into cellular dysfunction, death, and tissue fibrosis, establishing it as a key component in the multifactorial pathogenesis of RIHD.

### 3.4 DNA Damage and Cellular Senescence

Radiation causes double-strand breaks (DSBs) and other forms of DNA damage in cardiovascular cells [52]. While DNA repair pathways (*e.g*., NHEJ, HR) are activated, persistent or misrepaired lesions can trigger cellular senescence, particularly in endothelial and smooth muscle cells [53]. Senescent cells secrete the SASP factors, which propagate tissue dysfunction in a paracrine manner [45]. Accumulation of senescent cells in irradiated hearts contributes to chronic inflammation and fibrosis. Activation of cardiac fibroblasts into myofibroblasts driven by TGF-β, angiotensin II, and ROS leads to excessive deposition of extracellular matrix (ECM) proteins such as collagen I and III [54]. This replacement fibrosis disrupts myocardial architecture, impairs electrical conduction, and reduces ventricular compliance, ultimately causing diastolic dysfunction and heart failure with preserved ejection fraction (HFpEF) [55]. Radiation also promotes endothelial-to-mesenchymal transition (EndMT), further expanding the pool of collagen-producing cells [56]. Study reports that tRF-Glu-CTC is upregulated following vascular injury and suppresses fibromodulin expression in vascular smooth muscle cells [57].

## 4 tsRNAs IN CARDIOVASCULAR PHYSIOLOGY AND PATHOLOGY

tsRNAs have emerged as critical regulators in cardiovascular biology, operating at the interface between cellular homeostasis and stress response. At present, tsRNAs are recognized as stable, abundant, and functionally diverse non-coding RNAs that modulate gene expression at transcriptional, post-transcriptional, and translational levels. Their functions range from maintaining cardiac and vascular integrity under physiological conditions to driving pathological remodeling in cardiovascular diseases.

### 4.1 Roles of tsRNAs in Cardiovascular Functions

While the pathological roles of tsRNAs have been widely explored, emerging evidence suggests they also contribute to the maintenance of cardiovascular homeostasis [10]. In healthy cardiomyocytes and vascular cells, tsRNAs are involved in regulating essential cellular processes such as energy metabolism, mitochondrial function, and redox balance [58, 59]. In the context of radiation-induced heart injury, tsRNA dysregulation is implicated across the multifactorial pathogenesis of RIHD. Apart from canonical mechanisms of DNA damage and oxidative stress, radiation induces persistent ER stress, disrupts mTOR signaling and autophagic flux, and triggers chronic inflammatory responses, all of which converge to promote cardiomyocyte apoptosis, endothelial dysfunction, and fibrotic remodeling [38, 40]. tsRNAs have emerged as key modulators within these pathways, capable of both protective and deleterious effects depending on cellular context and stressor. For example, under ischemic stress, tRF5-22-SerGCT-1 is upregulated after myocardial infarction and exerts a protective effect by targeting MSK1 to modulate cardiomyocyte apoptosis [60]. Similarly, tRF-16-R29P4PE is downregulated in pathological cardiac hypertrophy (PCH), while its overexpression significantly enhances mitochondrial membrane potential, ATP production, and glucose levels, and alleviates disease progression by targeting PACE4 to modulate the HIF-1α/PPARα signaling pathway [61]. Meanwhile, tsr007330 is downregulated after MI and attenuates myocardial fibrosis by antagonizing NAT10-mediated EGR3 mRNA acetylation [62]. Conversely, the aberrant expression of specific tsRNAs directly drives cardiac injury. M1-macrophage-derived extracellular vesicles (EVs) deliver tsRNA-5006c to promote aortic valve interstitial cell (AVIC) osteogenesis *via* mitophagy regulation, highlighting a key mechanism in calcific valve disease [63]. tsRNA-5008a is significantly upregulated in atrial tissue during atrial fibrillation, where it directly suppresses SLC7A11 expression, leading to impaired glutathione metabolism, decreased GPX4 activity, iron accumulation, lipid peroxidation, and ultimately cardiomyocyte ferroptosis, thereby promoting atrial fibrosis and arrhythmogenesis [64].

A pivotal link between tsRNA dysregulation and radiation-induced mitochondrial injury involves the suppression of peroxisome proliferator-activated receptor gamma coactivator 1-alpha (PGC-1α). PGC-1α is a master regulator of mitochondrial biogenesis and antioxidant defense. Its downregulation is a hallmark of metabolic and oxidative stress in the heart. The tsRNA 5'tiRNA-33-CysACA-1, which is upregulated in septic cardiomyopathy, directly targets PGC-1α mRNA to reduce its stability, thereby suppressing mitochondrial biogenesis and exacerbating cardiac injury [64]. This mechanism is highly relevant to RIHD, where radiation-induced oxidative stress and energetic deficit are central. The suppression of PGC-1α, potentially mediated by specific stress-induced tsRNAs like 5'tiRNA-33-CysACA-1, would create a vicious cycle: impaired mitochondrial biogenesis compromises ATP production and antioxidant capacity, which amplifies oxidative stress and further damages mitochondria. This positions tsRNA-mediated repression of PGC-1α as a plausible and potent mechanism contributing to the sustained mitochondrial dysfunction observed in chronic radiation injury. Collectively, in cardiovascular biology, these findings highlight tsRNAs as critical regulators of physiological homeostasis under normal conditions and as key drivers of pathological remodeling when dysregulated. Their ability to interface with core injury pathways, including mitochondrial biogenesis *via* PGC-1α, positions them as promising diagnostic markers and potential therapeutic targets for a wide range of cardiovascular diseases, including radiation-induced heart disease.

### 4.2 tsRNAs in Radiation Response and DNA Damage

Emerging evidence indicates that tsRNAs are pivotal regulators not only involved in cardiovascular and metabolic regulation but also in cellular response to genotoxic stress, particularly radiation and DNA damage [65]. For example, ionizing radiation induces dysregulation of tsRNAs in human bronchial epithelial cells. Among these, tRF-Gly-GCC has been identified as a critical mediator that promotes oxidative stress, ROS production, and apoptosis potentially *via* PI3K/AKT and FOXO1 signaling, thus implicating tsRNAs in radiation-induced DNA damage response and lung injury pathogenesis [66]. Similarly, tRF-16-7X9PN5D is significantly downregulated in radioresistant colorectal cancer cells. Its expression enhances radiosensitivity by directly targeting MKNK1 to suppress the MKNK1-eIF4E signaling axis, thereby inhibiting proliferation, migration, invasion, and radiation resistance. This highlights a novel tsRNA-mediated regulatory pathway in the DNA damage response and radiotherapy resistance [67].

Notably, circulating tsRNA have also been demonstrated to serve as novel radiation biomarkers following whole-body irradiation with X-rays, protons, or carbon ions. These biomarkers are reproducible across mouse strains and detectable in humans, enabling rapid, sensitive, and specific non-invasive radiation biodosimetry within 4 h [68]. Upon exposure to radiation, global tRNA cleavage is rapidly induced, leading to abundant production of specific tsRNAs, including 5’-tiRNAs and 3’-tiRNAs, which function as signaling molecules in the DNA damage response (DDR) network [69]. 5'-tiRNA-His-GTG is upregulated in skin photoaging and promotes cellular senescence by targeting NUP98 to activate the JNK signaling pathway, and its inhibition ameliorates UVB-induced photoaging *in vitro* and *in vivo* [70]. In addition, 5’-tiRNA-Glu-TTC is upregulated by UVB exposure and promotes skin photoaging by directly targeting TRPV3 to activate the PI3K/AKT signaling pathway, with its inhibition alleviating photoaging phenotypes *in vitro* and *in vivo*. These findings highlight a novel tsRNA-mediated mechanism in radiation-like stress responses and tissue aging [71].

## 5 MECHANISTIC LINKS

Emerging evidence places tsRNAs at the epicenter of radiation-induced pathophysiology, as their biogenesis is robustly induced by ionizing radiation, a potent trigger of DNA damage, oxidative stress, ER stress, and cellular senescence [72]. Crucially, tsRNAs do not merely respond to these stresses; they actively orchestrate and amplify the key pathological cascades that define RIHD. Their functional convergence spans multiple, interconnected pathways, positioning them as central molecular transducers that convert acute genotoxic stress into chronic cardiovascular injury.

### 5.1 Amplification of Core Stress Responses

Radiation-induced tsRNAs critically modulate oxidative stress-mitochondrial dysfunction axis, a pivotal early node in RIHD. They exacerbate oxidative burden by suppressing antioxidant defenses (*e.g*., SOD2, Nrf2) and directly interfering with mitochondrial quality control. For instance, certain tsRNAs can target transcripts involved in mitochondrial biogenesis (*e.g*., PGC-1α) or mitophagy, thereby sustaining ROS production and energetic failure [42, 73]. Furthermore, emerging data suggest that tsRNAs are implicated in regulating the ER stress-mTOR-autophagy axis. By potentially targeting components of the UPR or autophagic machinery, specific tsRNAs may modulate the critical switch from adaptive proteostasis to maladaptive autophagy and cell death, thereby contributing to endothelial and cardiomyocyte loss [38, 40].

### 5.2 Propagation of injury and remodeling

Beyond modulating primary stress responses, tsRNAs directly drive downstream pathological processes. They amplify chronic inflammation by modulating signaling pathways such as PI3K/AKT and JNK, thereby increasing expression of pro-inflammatory cytokines like NF-κB, IL-6, and TNF-α [36]. Concurrently, tsRNAs promote endothelial dysfunction and microvascular rarefaction, impairing tissue perfusion. They also influence cardiac fibroblast activation and fibrotic remodeling, and drive cellular senescence and the SASP through pathways involving p53, p16, and NF-κB [70].

### 5.3 Integrated Role as Central Mediators

Critically, as radiation itself is a potent inducer of tsRNA generation, these molecules form a feed-forward signaling network. They are both products of radiation stress and active drivers that propagate and interconnect damage across cellular compartments (nucleus, mitochondrion, ER) and cell types (cardiomyocytes, endothelium, fibroblasts, immune cells) [73]. This positions tsRNAs not as bystanders but as central integrators that connect initial DNA injury with the sustained oxidative, inflammatory, fibrotic, and senescent responses, ultimately leading to long-term cardiovascular dysfunction. Their presence in circulation further underscores their role in systemic signal amplification.

## 6 CHALLENGES AND FUTURE DIRECTIONS

Despite growing recognition of tsRNAs as key players in radiation-induced cardiovascular injury, several challenges still remain. First, a lack of standardized nomenclature hampers cross-study comparisons. Inconsistent naming conventions (*e.g*., as tRF-5, tiRNA, and 5′-tsRNA) can lead to misannotation. Therefore, adopting a unified database such as MINTbase or tRFdb is strongly recommended to ensure consistent tsRNA identification. Second, most functional studies rely on *in vitro* gain- or loss-of-function experiments using synthetic mimics or inhibitors, which are limited by off-target effects and transient expression. A major gap is the lack of *in vivo* genetic models (*e.g*., transgenic or knockout) targeting individual tsRNAs, leaving causal relationships incompletely established. Third, target validation remains challenging because tsRNAs may act through non-canonical mechanisms beyond miRNA-like RNA interference, such as direct RNA-RNA interactions, RNA-protein binding, and modulation of stress granule dynamics. Finally, there is a critical paucity of data on the dose-response relationship and temporal dynamics of tsRNA expression across different radiation qualities (X-rays, protons, heavy ions), which limits their utility in biodosimetry and predictive modeling.

To advance the field, future research should focus on several key priorities. First, comprehensive radiotranscriptomic profiling with time- and dose-resolved small RNA sequencing is needed to map dynamic tsRNA expression in cardiovascular tissues after radiation. Second, applying single-cell and spatial transcriptomics can develop cell-type-specific tsRNA models in cardiomyocytes, endothelial cells, fibroblasts, and immune cells, revealing their niche-specific roles in radiation injury. Third, tsRNAs are promising therapeutic targets; antisense oligonucleotides (ASOs) or small molecules could be designed to inhibit pathogenic species, such as pro-senescence or pro-fibrotic tsRNAs. In addition to directly targeting tsRNAs, modulating their upstream regulatory networks or downstream effectors offers complementary therapeutic avenues. As discussed, the mTOR pathway, particularly mTOR complexes 1 (mTORC1) and 2 (mTORC2), is a critical node that integrates radiation-induced ER stress, autophagy, and fibrotic signaling. Emerging preclinical evidence suggests that novel allosteric or ATP-competitive mTOR inhibitors, or agents that selectively modulate mTORC1 versus mTORC2 activity, hold promise for mitigating cardiac remodeling in various diseases [74, 75]. Hence, investigating whether such mTOR-regulating molecules can ameliorate tsRNA-mediated damage or disrupt the pathogenic tsRNA-mTOR axis might also contribute to future research in RIHD. In parallel with pharmacological interventions, technological advances in radiotherapy are crucial for primary prevention. FLASH radiotherapy, which delivers radiation at ultra-high dose rates (>40 Gy/s), has emerged as a groundbreaking modality. Preclinical studies demonstrate that FLASH proton or electron therapy can significantly spare normal tissues, including the heart, from acute and late toxicities, with marked reductions in inflammation, endothelial dysfunction, and fibrosis compared to conventional dose-rate irradiation [76, 77]. Therefore, integrating tsRNA tsRNA studies under FLASH conditions is essential to decipher the molecular basis of this “FLASH effect” and to determine whether the tsRNA dysregulation profile is fundamentally altered, potentially contributing to the identification of novel protective mechanisms. Finally, translation to the clinical setting is paramount. Large-scale, multi-center prospective studies are essential to validate circulating tsRNAs as predictive biomarkers for radiation-associated cardiovascular events in cancer survivors. Coupling tsRNA profiling with advanced imaging and clinical phenotyping will enable better risk stratification and guide timely preventive interventions. In conclusion, overcoming the current challenges requires a multi-pronged strategy. This entails deepening the mechanistic understanding of tsRNAs through advanced omics, exploring their therapeutic potential directly and *via* pathway modulation (*e.g*., mTOR), leveraging technological breakthroughs like FLASH radiotherapy to reduce injury at its source, and rigorously validating findings in clinical cohorts. Integrating tsRNA biology into the evolving landscape of radiation medicine and cardio-oncology is crucial for improving the long-term cardiovascular outcomes of cancer patients.

## CONCLUSION

In summary, tsRNAs could be considered as critical molecular bridges linking radiation stress to cardiovascular pathology. Their rapid and specific induction following irradiation positions them as promising early biomarkers for non-invasive early detection and risk prediction of radiation-induced cardiovascular injury. Beyond diagnostics, pathogenic tsRNAs are now recognized as active drivers of oxidative stress, inflammation, fibrosis, and cellular senescence. This functional role highlights their potential as novel therapeutic targets, suggesting that modulating their expression or function may delay or even reverse radiation-associated tissue damage. Realizing this potential requires collaborative efforts across disciplines, integrating radiation biology, cardiovascular medicine, and RNA biology to decode tsRNA mechanisms, validate clinical applications, and develop targeted interventions. Such interdisciplinary synergy is essential to advance radiation protection strategies and improve long-term outcomes for cancer survivors.

## LIST OF ABBREVIATIONS

tsRNA: tRNA-derived small RNA
tRF: tRNA-derived fragment
tiRNA: tRNA-derived stress-induced RNA
ROS: Reactive oxygen species
ANG: Angiogenin
AGO: Argonaute
RIHD: Radiation-induced heart disease
